# Superiority of coarse eggshell as a calcium source over limestone, cockle shell, oyster shell, and fine eggshell in old laying hens

**DOI:** 10.1038/s41598-021-92589-y

**Published:** 2021-06-24

**Authors:** Woo-Do Lee, Damini Kothari, Kai-Min Niu, Jeong-Min Lim, Da-Hye Park, Jaeeun Ko, Kidong Eom, Soo-Ki Kim

**Affiliations:** 1 Department of Animal Science and Technology, Konkuk University, 120 Neungdong-ro, Gwangjin-gu, Seoul, 05029 Republic of Korea; 2 Institute of Biological Resource, Jiangxi Academy of Sciences, Nanchang, 330029 China; 3 Department of Veterinary Radiology and Diagnostic Imaging, College of Veterinary Medicine, Konkuk University, 120 Neungdong-ro, Gwangjin-gu, Seoul, 05029 Republic of Korea

**Keywords:** Biochemistry, Zoology

## Abstract

Chicken eggshell (ES) waste is a rich source of calcium carbonate (CaCO_3_); however, the potential of ES as dietary calcium (Ca) in old laying hens has not been explored. This study compared the effects of feeding limestone, cockle shell, oyster shell, fine ES, and coarse ES as the sole Ca source on production performance, egg quality, blood biochemical constituents, and tibia characteristics in old laying hens. A total of 450 ISA-Brown laying hens at 73 wk of age with similar egg production rate (EPR) were randomly assigned to 5 treatment groups (90 hens/group, 9 hens/replicate) for 7 wk. Dietary treatment groups comprised a corn-soybean meal based diet containing different Ca sources: (i) limestone (LS; < 2 mm and 2–4 mm mixed in the ratio of 3:7) as control, (ii) cockle shell (CS; 1–4 mm), (iii) oyster shell (OS; 3–16 mm), (iv) ES fine particles (ESF; < 1 mm), and (v) ES coarse particles (ESC; 3–5 mm). Results indicated that dietary inclusion of coarse ES particles significantly increased average egg weight (*P* < 0.001) and daily egg mass (*P* < 0.05), and decreased feed conversion ratio (*P* < 0.001) as compared with the other treatments. However, no significant differences in EPR, feed intake, cracked egg proportion, and mortality were observed among the dietary treatments (*P* > 0.05). Notably, the use of ESF led to a lower proportion of cracked eggs than ESC (*P* < 0.05). ESC fed hens produced the heaviest eggs whereas CS fed hens produced the lightest (*P* < 0.001); the particle size of ES also affected the egg weight (*P* < 0.05). The eggs from OS and ESC fed hens showed a greater albumen height in comparison to eggs from CS group (*P* < 0.05); but no significant difference was observed among the LS, OS, ESF, and ESC groups (P > 0.05). The yolk color was darker in the eggs of group ESF as compared with other dietary groups (*P* < 0.01). However, no significant effects on Haugh units and shell properties were observed among the treatments (*P* > 0.05). The blood biochemistry results were not affected by the dietary Ca (*P* > 0.05) except for lower levels of high-density lipoprotein percentage (HDL %) in OS and ESC fed hens (*P* < 0.05). The tibia characteristics including weight, length, width, and breaking strength did not differ among the dietary groups (*P* > 0.05). However, the ESC and OS fed hens showed higher tibia bone mineral density (BMD) than the other groups (*P* < 0.001). In conclusion, coarse ES as a sole Ca source had beneficial effects on the production performance, egg quality, and tibia BMD in old laying hens.

## Introduction

Feed constitutes approximately 60–70% of the total cost of poultry production^1^. In recent years, the use of unconventional feed resources which are cheap, widely available, and relatively high in nutrient content, as well as reducing environmental problems have become increasingly popular. Chicken eggshell (ES), a major poultry by-product, is largely considered as a hazardous solid waste under the EU legislation and approximately 110 billion tons of ES were wasted in 2016 globally^2^. In Korea, about 90,000 tons of ES waste are produced every year^3^. Currently, most of the ES waste without any pre-treatment is transported to countryside landfill at a high management cost^4^. ES contains more than 96% calcium carbonate (CaCO_3_), so it could act as a potential substitute for natural mined limestone^4,5^. ES represents about 40% of elemental calcium (Ca) and contains about 385–401 mg Ca per gram^6,7^. Generally, laying hens require an average of 4 to 4.5 g of Ca supplementation every day^8^ and, therefore, approximately 12 g of ES could provide their daily Ca need. Consequently, for every thousand tons of ES waste, 400 tons of Ca could be produced to feed approximately 80 million laying hens daily. This would reduce the cost of feed as well as increase the sustainability of poultry production.

In efforts to reduce the widespread occurrence of ES waste, several researchers investigated the potential of ES as a Ca substitute in laying hen diets. Muir et al.^9^ reported no adverse effect of ES supplementation on egg production, body weight, egg weight, and eggshell thickness in laying hens during 32 to 72 wk of age. Scheideler^10^ indicated the high availability of Ca from ground ES to support egg production in laying hens but recommended that it should be combined with a large-particle Ca source to support optimal eggshell quality. Gongruttananun^11^ also reported that ground ES can be fully used as a Ca source in the first-cycle layer diets without negative effects on productive traits, egg and eggshell quality, serum Ca balance, bone mineralization, and gonadal performance. Apart from the Ca source, the particle size of Ca sources may also influence its availability to the hens^12–15^. However, the particle size of ES was not considered in these previous studies. Also, the age-related decline in eggshell and bone quality is another major concern in the poultry industries^16,17^ pertaining to the lower efficiency of Ca absorption in old laying hens^14,18–20^. In this context, Lichovnikova^13^ recommended that diets of old laying hens should contain two-thirds large particle Ca to achieve good eggshell quality.

Mitigation of food waste is a step toward attaining global environmental goals^21^. Keeping this in mind, we focused on the utilization of ES for laying hen nutrition which may reduce the global burden of ES waste to a certain extent. This study was designed to evaluate the effects of ES in different particle sizes (< 1 mm and 3–5 mm), completely replacing limestone, on productive performance, egg quality, blood biochemical constituents, and tibia characteristics of laying hens during the late laying period. Other commonly used supplemental Ca sources in Korea were included for the purposes of comparison.

## Results

### Production performance

The effects of different Ca sources on laying hens’ productivity are shown in Table 1. The laying performance of the birds was lower than breed standard^22^ for the ISA-Brown layers in this study with respect to their age period, which could be explained by lower egg production rate (EPR) at the start of experiment (data not shown) pertaining to the high environmental temperature (30–34℃). There was no difference among the treatments in EPR, feed intake (FI), cracked eggs, and mortality during the 7-week experimental period (*P* > 0.05). However, the average egg weight (AEW) was affected by the source of Ca (*P* < 0.001), with the ES coarse particles (ESC; 3–5 mm) fed group producing the heaviest eggs followed by ES fine particles (ESF; < 1 mm), limestone (LS; < 2 mm and 2–4 mm mixed in the ratio of 3:7), oyster shell (OS; 3–16 mm), and cockle shell (CS; 1–4 mm) fed groups. The OS and ESC treatments increased the daily egg mass as compared to the control (LS) (*P* < 0.05), which contributed to an improvement in feed conversion ratio (FCR) in the OS and ESC groups (*P* < 0.05). Considering the effect of ES particle size, it was observed that ESC treatment had significantly improved AEW, daily egg mass, and FCR as compared with ESF (*P* < 0.05); however, the proportion of cracked eggs was lower in ESF than ESC (*P* < 0.05).

**Table 1:** Overall performance of laying hens according to the dietary treatments between 73 and 80 week of age.

Items	Treatments					SEM	*P*-value	
	LS	CS	OS	ESF	ESC		Source	ES size
EPR, %	52.40	57.60	55.69	54.04	57.03	1.27	0.903	0.619
Average egg weight, g	65.06^b^	63.20^c^	64.87^b^	65.21^b^	65.93^a^	0.186	< 0.001	0.049
Daily egg mass, g/hen/day	34.07^c^	36.45^ab^	36.17^ab^	35.21^bc^	37.64^a^	0.350	0.011	0.017
FI, g/hen/day	106.3	107.3	105.6	106.4	106.5	0.795	0.979	0.964
FCR, g feed/g egg	3.26^a^	3.21^ab^	3.05^bc^	3.27^a^	2.90^c^	0.035	< 0.001	< 0.001
Cracked egg proportion, %	1.69	1.69	1.99	1.41	2.53	0.160	0.222	0.029
Mortality, %	5.56	6.67	6.67	3.33	3.33	0.157	0.781	1.000

Results are the means of 10 replicates of 9 hens each per treatment for the 7-wk experimental period.

*LS* limestone (< 2 and 2–4 mm mixed in 3:7); *CS* cockle shell (1–4 mm); *OS* oyster shell (3–16 mm); *ESF* eggshell fine particles (< 1 mm);* ESC* eggshell coarse particles (3–5 mm).

*SEM* standard error of means, *ES* eggshell, *EPR* egg production rate, *FI* feed intake, *FCR* feed conversion ratio.

^a-c^Means with the different superscript in the same row differ significantly (*P* < 0.05).

### Egg quality

The average egg quality traits for a period of 7 week are presented in Table 2. Hens fed ESC produced the heaviest eggs whereas CS fed hens produced the lightest (*P* < 0.001); the particle size of ES also affected the egg weight (*P* < 0.05) during the 7-week experimental period. Dietary Ca sources had a significant effect on albumen height with the eggs obtained from OS and ESC fed hens having greater albumen height than CS group (*P* < 0.05); however, no significant difference was observed among the LS, OS, ESF, and ESC groups (*P* > 0.05). The yolk color measurements also differed significantly among the dietary treatments (*P* < 0.01), whose highest value was recorded in the ESF group and the lowest in eggs from the CS group. Nevertheless, the ES particle size had no significant effect on albumen height and yolk color (*P* > 0.05). Furthermore, there was no difference in Haugh units and eggshell properties (color, strength, and thickness) among the dietary treatments during the experiment (*P* > 0.05).

**Table 2:** Egg quality traits of laying hens according to the treatments between 73 and 80 wk of age.

Items	Treatments					SEM	*P*-value	
	LS	CS	OS	ESF	ESC		Source	ES size
Egg weight, g	65.40^b^	63.15^c^	64.93^b^	65.43^b^	66.30^a^	0.214	< 0.001	0.035
Haugh units	84.85	83.60	86.06	84.90	85.98	0.313	0.072	0.249
Albumen height, mm	7.40^ab^	7.14^b^	7.65^a^	7.50^ab^	7.62^a^	0.060	0.035	0.457
Egg shell color	34.98	34.40	34.83	34.23	34.30	0.346	0.952	0.953
Egg yolk color	5.78^bc^	5.71^c^	5.75^bc^	5.97^a^	5.88^ab^	0.026	0.004	0.212
Egg shell breaking strength, kgf	3.62	3.79	3.72	3.76	3.76	0.025	0.252	0.837
Egg shell thickness, mm	0.392	0.393	0.393	0.383	0.389	0.002	0.636	0.460

Results are the means of randomly sampled 280 eggs per treatment (40 eggs per treatment at each week; 4 eggs per replicate) during 73 to 80 week of age.

*LS* limestone (< 2 and 2–4 mm mixed in 3:7); *CS* cockle shell (1–4 mm); *OS *oyster shell (3–16 mm); *ESF* eggshell fine particles (< 1 mm), *ESC* eggshell coarse particles (3–5 mm).

*SEM* standard error of means, *ES* eggshell.

^a-c^Means with the different superscript in the same row differ significantly (*P* < 0.05).

### Blood biochemical constituents

No statistically significant changes were observed in the blood biochemical constituents among the dietary treatments (*P* > 0.05), except for high density lipoprotein (HDL) (%) (Table 3). The HDL proportion showed a decreasing trend in response to dietary ESC and OS supplementation as compared with LS, ESF, and CS treatments (*P* < 0.01).

**Table 3:** Blood biochemical constituents of laying hens at 80 week of age according to the treatments.

Items	Treatments					SEM	*P*-value	
	LS	CS	OS	ESF	ESC		Source	ES size
AST, U/L	184.7	183.1	171.41	178.3	194.2	5.22	0.752	0.359
ALT, U/L	6.58	4.25	5.67	5.17	8.75	0.732	0.365	0.126
LDH, mg/dl	6749.2	7511.7	6414.2	5594.2	5594.2	309.7	0.403	0.506
TG, mg/dl	2464.2	1900.0	2644.2	3084.2	3132.5	171.7	0.138	0.928
TC, mg/dl	134.0	103.8	138.1	150.3	157.8	6.53	0.084	0.707
HDL, mg/dl	40.00	32.42	33.92	39.00	36.58	1.09	0.128	0.472
HDL, %	32.09^a^	32.70^a^	24.53^b^	27.38^ab^	24.87^b^	0.959	0.007	0.367
LDL + VLDL, mg/dl	94.00	71.42	104.2	111.3	121.2	5.78	0.065	0.573
Glucose, mg/dl	267.3	280.6	282.9	269.7	272.0	7.53	0.961	0.926
TP, g/dl	6.81	6.03	6.78	6.77	7.11	0.122	0.068	0.359
Albumin, g/dl	2.43	1.98	2.03	2.13	2.65	0.092	0.092	0.068
Creatinine, mg/dl	0.250	0.250	0.267	0.242	0.275	0.014	0.947	0.471
BUN, mg/dl	2.44	2.40	2.41	2.64	2.53	0.047	0.447	0.471

Results are the means of 12 hens at 80 week of age.

*LS* limestone (< 2 and 2–4 mm mixed in 3:7); *CS* cockle shell (1–4 mm); *OS* oyster shell (3–16 mm); *ESF* eggshell fine particles (< 1 mm); *ESC* eggshell coarse particles (3–5 mm).

*SEM *standard error of means, *ES* eggshell, *AST* aspartate aminotransferase, *ALT *alanine aminotransferase, *LDH* lactate dehydrogenase, *TG *triglycerides, *TC* total cholesterol, *HDL *high density lipoprotein, *LDL* low density lipoprotein, *VLDL *very low density lipoprotein, *TP *total protein, *BUN* blood urea nitrogen (BUN).

^a,b^Means with the different superscript in the same row differ significantly (*P* < 0.05).

### Serum Ca and P levels

**Figure 1:**
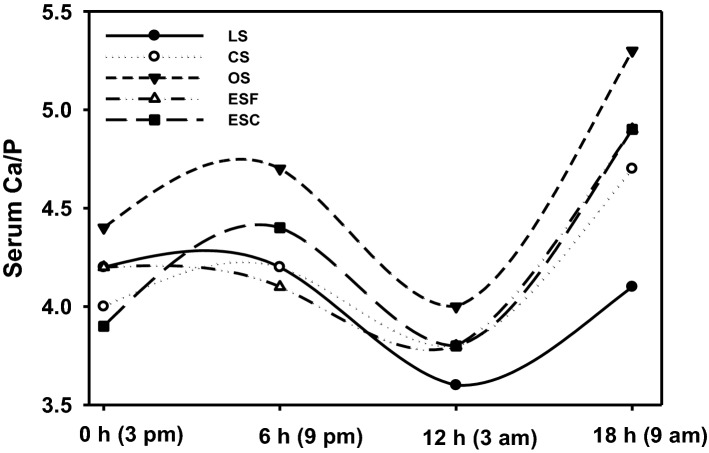
Effects of different dietary Ca sources on the serum Ca and P ratio during light and dark periods (18 h) in laying hens at 80 week of age. *LS* limestone (< 2 and 2–4 mm mixed in 3:7); *CS* cockle shell (1–4 mm); *OS* oyster shell (3–16 mm); *ESF* eggshell fine particles (< 1 mm); *ESC* eggshell coarse particles (3–5 mm). Results are the means of 4 hens per treatment.

The analysis of serum Ca and P levels during light and dark periods provides valuable information about the daily Ca utilization in hens. There was no significant effect on the Ca to P ratio in the serum of hens fed different dietary Ca sources during light and dark periods (Fig. 1). At the first sampling time (3 pm), when the hens consumed sufficient feed, there was no significant effect of the different Ca dietary sources on the Ca to P ratio. When the lights were turned off (9 pm), the serum Ca to P ratio increased in all the treatment groups, indicating efficient Ca absorption from different Ca sources. At 3 am (in this study, oviposition occurred at 3 am-10 am), the serum Ca levels decreased while P levels increased (Supplementary Table S3), resulting in the lower serum Ca to P ratio in case of all the treatment groups, which indicated the use of all dietary Ca sources for shell formation. In the next light period (9 am), when the birds started eating again, the serum Ca to P ratio replenished, implying the absorption of Ca from the different dietary sources.

### Tibia characteristics

There were no significant differences in weight, length, width, and bone breaking strength of tibiae among the dietary treatments (Table 4). However, significant differences in the tibia bone mineral density (BMD) among treatments were observed at the end of the experimental period (Table 4). The tibia neck of LS, OS, and ESC fed hens had higher BMD as compared with CS and ESF fed hens (*P* < 0.001). In addition, significantly higher BMD in proximal and distal tibia regions as well as in total tibia were observed in OS and ESC fed hens as compared with other treatments (*P* < 0.001). Furthermore, ESC treatment had higher tibia BMD as compared with ESF treatment (*P* < 0.001; *P* < 0.05), indicating higher mineralization of dietary Ca in the bones.

**Table 4:** Tibia bone quality traits of laying hens at 80 week of age according to the treatments.

Items	Treatments					SEM	*P*-value	
	LS	CS	OS	ESF	ESC		Source	ES size
Bone weight, g	12.33	11.83	11.98	11.92	12.31	0.141	0.746	0.400
Bone length, mm	118.1	116.8	118.7	117.7	118.4	0.452	0.743	0.658
Bone width, mm	7.20	7.13	7.47	7.67	7.33	0.077	0.218	0.185
Bone breaking strength, kgf	23.95	23.97	23.12	23.95	25.47	0.743	0.917	0.542
**BMD, mg/cm**^**3**^								
Tibia neck	276.1^a^	226.5^b^	295.4^a^	219.1^b^	309.5^a^	8.11	< 0.001	< 0.001
1/3 tibia	302.2^b^	284.8^b^	378.2^a^	261.1^b^	384.7^a^	9.13	< 0.001	< 0.001
2/3 tibia	340.8^b^	322.6^b^	463.4^a^	363.9^b^	452.4^a^	12.21	< 0.001	0.016
Total BMD	306.4^b^	278.0^b^	379.0^a^	281.4^b^	382.2^a^	8.63	< 0.001	< 0.001

Results are the means of 16 hens at 80 wk of age.

*LS* limestone (< 2 and 2–4 mm mixed in 3:7); *CS* cockle shell (1–4 mm), *OS* oyster shell (3–16 mm); *ESF* eggshell fine particles (< 1 mm); *ESC* eggshell coarse particles (3–5 mm).

*SEM* standard error of means, *ES* eggshell, *BMD *bone mineral density.

^a,b^Means with the different superscript in the same row differ significantly (*P* < 0.05).

## Discussion

Several studies have supported the nutritional value of ES waste as a possible alternative Ca source for laying hens^9–11^. However, the effects of different particle sizes of ES on the production performance, egg quality, blood biochemical profile, and tibia characteristics in old laying hens have not been investigated yet. In the present study, EPR was not different among the groups, suggesting a similar dietary efficiency of all the Ca sources. These results are in line with several findings^10,11,23,24^, which indicated non-significant effects of the Ca source on egg productivity in laying hens. In contrast, Ahmad and Balander^25^ indicated improved egg productivity when 50% of limestone in the diet was replaced by oyster shell. Over the course of entire experiment (73 to 80 week), the feed consumption was not affected, indicating all the Ca sources used in this study were able to provide sufficient Ca to the hens. The improvements in AEW, daily egg mass, and FCR in the ESC and OS fed groups observed in this study are most likely due to their larger particle size. Large particles of Ca are reported to stay longer in the gizzard because of their slow passage in the gastrointestinal tract, which in turn increases in vivo solubility and availability as compared with those of fine particles^26,27^. Although, the literature supporting the improved performance traits with ES with different particle sizes in laying hens is scarce, only one study recommended that ES product when used as a Ca supplement in layers’ diet should not be ground^10^. Nonetheless, there are several studies that investigated the effect of inclusion of limestone or oyster shell with different particle sizes on laying hens’ productivity^12,14,23,28–30^. For example, Saunders-Blades et al.^29^ indicated that feeding large particle limestone decreased bone resorption in laying hens and thereby had beneficial effects on the birds’ health and production quality. Skřivan et al.^30^ demonstrated that feeding younger and older hens a Ca source with large particles (0.8–2 mm) of limestone increased egg production, egg weight, and FCR. However, the longer retention of a coarse Ca source may sometimes induce negative effects in old laying hens due to its inability to supply sufficient amount of Ca for initial stages of egg shell formation^31^. The intake of exclusively coarse ES particles in ESC group in this study might have caused lower availability of Ca for initial stages of shell formation, resulting in the increased percentage of cracked eggs as compared with the ESF group.

The dietary treatments did not affect most of the egg quality traits significantly, except for egg weight, albumen height, and yolk color. Guinotte and Nys^12^ indicated that layer hens fed larger limestone particles produced heavier eggs as compared with those consuming fine limestone particles. Egg shell quality is one of the major concerns while investigating the effects of different dietary Ca sources^29^. The Ca sources examined in this study, by completely replacing the limestone, did not induce any effects on egg shell quality (strength, thickness, and color). Previously, Olgun et al.^32^ also reported the inclusion of limestone, oyster shell and eggshell as Ca sources did not significantly affect the shell strength and thickness, in agreement with our results. Similarly, Ahmad and Balander^25^ reported no significant difference in the egg shell thickness when 50% of the limestone was replaced by oyster shell. In contrast, Lichovnikova^13^ recommended that two-thirds of the Ca source should be fed in the form of large particles (limestone grit or oyster shell) to ensure good egg shell quality in the last third cycle of the laying period. Likewise, Karunajeewa^33^ indicated the improvement in egg shell quality by cockle shell supplementation as compared with limestone. Our results might suggest that the requirement of laying hens to maintain egg shell quality was met by all the Ca sources used as per the recommendations of NRC^34^. Herein, the ES fed hen produced eggs with darker yellow yolk as compared with the other treatments. There is no information concerning the effect of ES or particle size of the ES on yolk color; thus, further studies are required to delineate the underlying mechanism.

The blood biochemical constituent analysis can be used to evaluate the metabolism of birds, thereby providing some indices for their health status^35^. The activities of aspartate aminotransferase (AST), alanine aminotransferase (ALT), and lactate dehydrogenase (LDH) are routinely used to evaluate liver health in laying hens^36,37^. In this study, the use of different Ca sources did not affect the serum ALT, AST, and LDH levels in laying hens, hence supporting their safety in laying hen diets on the liver metabolism. Serum proteins play a key role in body homeostasis and albumin serves as the most favorable source of amino acids for protein synthesis. Creatinine, along with blood urea nitrogen (BUN), are also indices of protein metabolism and kidney functions^38^. In our study, no differences in the levels of total proteins, albumin, creatinine, and BUN were observed among the treatment groups, indicating that Ca source had no effects on protein metabolism. Serum total cholesterol (TC) content has a major impact on egg yolk production in laying hens^39^ and it could be affected by various factors such as age, genetics, and nutrition, and typically ranges 100–200 mg/dl in laying hens^35^. In this study, TC remained unaffected among the dietary treatments; however, hens receiving ESC and OS containing diets presented a significant decrease in the HDL proportion as compared with LS, ESF, and CS fed groups. Nevertheless, the decreased proportion of HDL was within the normal range of HDL level, i.e. > 22 mg/dl, hence supports the safety of ESC or OS in lipid metabolism. Glucose levels are often used as indicators in determining insulin resistance and hyperglycemia^40^, remained unaffected at the end of experiment, indicating unaltered glucose metabolism by the Ca sources.

Ca and P are essential mineral nutrients in laying hens, as they play important roles in the bone and egg shell formation^41^. Ca is incorporated into bone as hydroxyapatite [Ca_5_(PO_4_)_3_(OH)], a P containing compound, and into egg shell as calcium carbonate (CaCO_3_). The metabolism of Ca and P is regulated by the dietary levels of each and through the synergistic actions of intestine, kidney, and skeleton^42,43^. Phosphorus, required in small amounts, reduces blood acidosis by flushing excess hydrogen ions through excretion and, hence, contributing to the maintenance of bicarbonate levels for egg shell formation^44^. However, when P is in excess, it binds with Ca in the intestine to form insoluble phosphate and interferes with the Ca absorption in the body, resulting in deterioration of egg shell quality. Conversely, P deficiency could induce Ca excretion, leading to false gait and leg abnormalities such as cartilage and cage scattering fatigue, so it is crucial to maintain a proper Ca to P ratio^44^. During an egg laying cycle, the serum Ca and P levels are changed in a dynamic manner^45,46^. Hens generally need 25–26 h to lay an egg, most of the time required for shell formation (18–20 h)^31,47^. Shell formation requires large amounts of Ca (2–2.5 g of Ca per egg), around 2/3^rd^ of which is supplied directly from the hen’s diet and the remaining 1/3^rd^ is mobilized from the medullary bone^48^. Shell formation occurs during the night period, the time when Ca levels are low in the gut since birds show nocturnal fast^49^. To establish the synchronization between the circadian availability of dietary Ca and the circadian deposition of Ca into the egg shell, two mechanisms were proposed: (a) efficient absorption of Ca during the early dark period, when feed is present in the gut; and (b) efficient bone resorption process during the later dark period^8^. In this study, the dietary inclusion of CS, OS, ESF, or ESC as a sole Ca source did not significantly affect serum Ca and P ratio before and after oviposition (*P* > 0.05) when compared with LS fed hens. Additionally, when the lights were turned off (9 pm), the serum Ca to P ratio was numerically higher in OS and ESC fed hens as compared with other groups, indicating the enhancement of Ca absorption for egg shell formation. Consequently, lower mobilization of Ca from medullary bones will occur for egg shell formation in the OS and ESC fed hens. This study’s results conjecture the advantage of using large particle Ca in layer’s diet, consistent with previous studies which indicate that the large particles may have slow passage through the gut because of their low solubility, thereby Ca being available to the hen for a longer period^24,30,31,50^.

The high Ca demands in laying hens during production to maintain structural bone quality have been well documented^51^. As birds grow older, bone mechanical properties and BMD gradually decline because of higher bone resorption and lower bone formation in the birds^52^. Additionally, the incomplete restoration of the circadian loss of bone Ca in the subsequent daylight period from feed results in poor bone quality in laying birds. In this study, supplementation of different Ca sources did not make any difference in bone weight and bone breaking strength of laying hens at late stage of production, indicating their ability to preserve the bone quality in old laying hens. In contrast, Meyer et al.^53^ reported higher bone strength in the hens fed oyster shell, eggshell, and limestone large particles than those consumed limestone small particles. Guinotte and Nys^12^ also indicated that the inclusion of large limestone or oyster shell led to improved tibial characteristics. QCT have been used to determine BMD in egg-laying hens^29,51^. The significantly increased BMD of total and individual tibia regions (proximal and distal) in the ESC and OS fed hens implied that less bone resorption might have occurred to support the egg production in old laying hens. This finding is consistent with the report of Saunders-Blades et al.^29^, which indicated that the inclusion of larger Ca particles (limestone) in diet improved tibia BMD in laying hens at 74 wk of age. Reports investigating the effect of different particle sizes of ES on tibia BMD are scarce in the available literature.

In conclusion, the complete substitution of limestone with ES provided sufficient Ca for performance, egg quality, and bone mineralization in old laying hens over the 7 week of study. In addition, ES with either fine or coarse particles did not show any adverse effects on biochemical constituents, and Ca and P levels in the serum of laying hens. The superiority of coarse ES particles was observed over the fine ES particles and may be attributed to their large particle size. However, the implications of long term feeding of the ESC containing diet to determine its efficacy in laying hens should be studied further. The application of ES waste as a commercial Ca source for laying hens could improve the economic efficiency while simultaneously reducing the environment burden of landfills.

## Methods

### Ethical statement

All experimental protocols were approved by the Animal Care and Use Committee (Approval number: KU18057) of Konkuk University (Seoul, Republic of Korea). Experiments were conducted at a private laying hen farm (Chung-ju, Republic of Korea) during August–October 2017 in accordance with the approved guidelines and regulations, and in compliance with the ARRIVE guidelines^54^.

### Experimental diets

Five different Ca sources including limestone (< 2 mm and 2–4 mm mixed in the ratio of 3:7; LS), cockle shell (1–4 mm; CS), oyster shell (3–16 mm; OS), ES fine particles (< 1 mm; ESF) and ES coarse particles (3–5 mm; ESC) were used in this study. Limestone and cockle shell were purchased from Seoul Feed Co., Ltd., (Seoul, Republic of Korea) and oyster shell was purchased from Jisan Industrial Co., Ltd. (Seoul, Republic of Korea). The fine and coarse particles of ES were produced and supplied by Poonglim Food Co., Ltd. (Seoul, Republic of Korea). Briefly, ES membranes were removed by washing with water followed by heating at 150℃ for 12 h, and then ES were crushed to a particle of 3–5 mm using a hammer mill (SM-D3, Wilhelm Siefer GmbH & Co., Velvert, Germany). ES particles of less than 1 mm were separated through a 1 mm sieve after further crushing.

A corn-soybean meal based diet was formulated to meet or exceed nutrient requirements of laying hens^34^. To the basal diet, each of the pre-analyzed Ca sources was added to achieve the desired concentration of Ca (4.1%) in the final diets. Cellulose was used as a filler in the experimental diets so that all diets could be formulated with various Ca sources at the same concentration. The Ca sources and cellulose were mixed thoroughly for 10 min in a feed mixer (DKM-350SU, Daekwang Machinery Co. Ltd., Hwaseong, Republic of Korea). All diets were isocaloric and isonitrogenous differing only in the ingredients used as the main Ca source. The ingredients and chemical composition of the experimental diets are shown in Table 5.

**Table 5:** Ingredients and nutrient composition of the experimental diets.

Items	Treatments				
	LS	CS	OS	ESF	ESC
**Ingredients, %**					
Corn	54.1	54.1	54.1	54.1	54.1
Soybean meal	23.6	23.6	23.6	23.6	23.6
Rapeseed meal	2.00	2.00	2.00	2.00	2.00
Distillers dried grains with solubles	6.00	6.00	6.00	6.00	6.00
Tallow	1.11	1.11	1.1	1.11	1.11
Molasses	0.500	0.500	0.500	0.500	0.500
Methionine (98%)	0.132	0.132	0.132	0.132	0.132
Dicalcium phosphate	0.510	0.510	0.510	0.510	0.510
Choline-Cl (liquid) 50%	0.100	0.100	0.100	0.100	0.100
Limestone, 37.2% Ca	10.5	–	–	–	–
Cockle shell, 37.5% Ca	–	10.4	–	–	–
Oyster shell, 36.39% Ca	–	–	10.7	–	–
Fine ES, 34.17% Ca	–	–	–	11.4	–
Coarse ES, 36.13% Ca	–	–	–	–	10.8
Cellulose	0.900	1.00	0.700	–	0.600
Salt	0.250	0.250	0.250	0.250	0.250
Sodium bicarbonate	0.060	0.060	0.060	0.060	0.060
Vitamin premix^a^	0.100	0.100	0.100	0.100	0.100
Mineral premix^b^	0.100	0.100	0.100	0.100	0.100
Phytase^c^	0.050	0.050	0.050	0.050	0.050
**Calculated nutrient composition, %**					
Crude protein	17.0	17.0	17.0	17.0	17.0
Crude fat	3.87	3.87	3.87	3.87	3.87
Crude fiber	3.39	3.48	3.27	2.69	3.19
Ash	14.8	14.8	14.8	14.8	14.8
Ca	4.11	4.10	4.09	4.01	4.10
Total P	0.430	0.441	0.477	0.499	0.490
Met + Cys	0.700	0.700	0.700	0.700	0.700
Available P	0.380	0.380	0.380	0.380	0.380
Metabolizable energy (MJ/kg)	11.4	11.4	11.4	11.4	11.4

*LS* limestone (< 2 and 2–4 mm mixed in 3:7); *CS *cockle shell (1–4 mm); *OS* oyster shell (3–16 mm); *ESF* eggshell fine particles (< 1 mm); *ESC* eggshell coarse particles (3–5 mm).

*Met* methionine, *Cys* cysteine.

^a^Vitamin premix supplied the following per kg of diet: vitamin A, 8000 IU; vitamin D3, 3300 IU; vitamin E, 20 g; vitamin K, 2.5 g; vitamin B1, 2.5 g; vitamin B2, 5.5 g; vitamin B3, 30 g; vitamin B5, 8 g; vitamin B6, 4 g; vitamin B7, 75 mg, vitamin B9, 0.9 g; vitamin B12, 23 mg.

^b^Mineral premix supplied the following per kg of diet: Choline, 110 g; Manganese, 90 g; Zinc, 80 g; Iron, 40 g; Copper, 8 g; Iodine, 1.2 g; Selenium, 0.22 g.

^c^1,000,000 phytase units (FTU).

### Birds and treatments

A total of 450 ISA-Brown hens at 71 week of age (1900.0 ± 271.4 g) were housed in two-stage metal cages (735 cm^2^/hen) under controlled conditions of temperature (23.6 ± 2.6 ℃) and humidity (76.4 ± 15.7%). After the 2-week adaptation on a corn-soybean meal based commercial diet (Supplementary Table S4), birds with the same egg production rate (EPR) were randomly assigned to 5 treatment groups with 10 replicates (cages) (nine birds per replicate/cage) and each cage was provided with three nest boxes. Group LS, serving as the control group, was provided with a layer diet comprising limestone as the Ca source, group CS was placed on a layer diet that contained cockle shell as the Ca source, group OS received a layer diet containing ground oyster shell as the Ca source, group ESF received a layer diet containing fine ES particles (< 1 mm) as the Ca source, and group ESC received a layer diet containing coarse ES particles (3–5 mm) as the Ca source. Feed in mash form was distributed manually once daily at 10 am. Feed and water were provided for ad libitum consumption*.* The birds were exposed to a total of 16 h of artificial photoperiod (16L:8D) daily from an automated lighting control system (SJP-E16 3 W, Seojun Electric Co. Ltd., Seoul, Republic of Korea) which was programmed to switch on between 5 am and 9 pm during the entire experiment.

### Hen productivity

The number of eggs laid by birds in each replicate was recorded daily once at 10 am and expressed as the percentage of egg production. The hen-day EPR is calculated by dividing the total number of eggs collected by the number of live hens daily in each replicate^55^. The total eggs produced in a day were weighed collectively for every replicate and was used to estimate average egg weight (AEW). Daily egg mass was calculated by multiplying EPR by AEW. Feed intake (FI) was measured weekly once per replicate, weighing the amount of feed distributed and that of residual and scattered feed. The feed conversion ratio (FCR) was calculated based on FI and daily egg mass^56^. The proportion of cracked eggs in each replicate was calculated weekly by dividing the number of cracked eggs by total eggs. Mortality was recorded during the experiment.

### Egg quality

Forty eggs per treatment (4 eggs per replicate) were randomly selected after each week and analyzed for their quality on the same day of collection. Egg quality traits including egg weight, albumen height, Haugh unit, yolk color, shell strength, and shell thickness were determined using an automatic egg analyzer (Digital egg tester DET6000, NABEL, Co. Ltd., Japan). The Haugh unit is calculated using the following equation: 100 × log (H + 7.57–1.7 × W^0.37^), where H = albumen height (mm) and W = egg weight (g)^57^. Shell color was determined by using a QCR shell color reflectometer (Technical Services and Supplies, York, UK) as indicated by Safaa et al.^23^. A total of 1400 eggs were analyzed throughout the 7 week of study.

### Blood sampling and analysis

At the end of the experiment, four hens (80 week of age) were randomly selected from each treatment at 3 pm, 9 pm and 3 am and designated for blood sampling. Blood samples were collected in Vacutainer Serum Tubes (BD, New Jersey, USA) from hens by cardiac puncture after CO_2_ euthanasia. Serum was collected by centrifugation at 1500 rpm for 10 min and stored at − 20 °C until analysis. Serum concentrations of aspartate aminotransferase (AST), alanine aminotransferase (ALT), blood urea nitrogen (BUN), lactate dehydrogenase (LDH), creatinine, glucose, total cholesterol (TC), high density lipoprotein (HDL) (mg/dl), HDL (% total), low density lipoprotein (LDL) + very low density lipoprotein (VLDL), total protein (TP), albumin and triglycerides (TG) were determined by using an automated clinical chemistry analyzer (FUJI DRI-CHEM 7000i, FUJIFILM Corporation, Japan). HDL (%) was expressed with the ratio of HDL to TC content, and LDL + VLDL was calculated by subtracting HDL from TC^58^.

To determine the serum Ca and P levels during light and dark periods, blood samples were collected at four time points (3 pm, 9 pm, 3 am, and 9 am) from four hens (80 week of age) per treatment as described above. Ca and P contents of the serum were measured using the automated clinical chemistry analyzer.

### Tibia characteristics

At the end of the experiment, 16 laying hens (80 week of age) per treatment group were randomly selected to collect right or left tibia after removing the non-bone tissues (fat, tendon, and muscle). The tibiae were individually sealed in plastic bags to minimize moisture loss and stored at 4 ℃ for one day. The length and width of the tibia was measured using a micrometer caliper and the weight was recorded. Tibia strength was determined from a 3-point bending test (ASAE Standards S459, 2001) using an Instron Universal Testing Machine (Model 3342, USA) at 50 kg load range and with a crosshead speed of 50 mm/min; tibia supported on a 3.35-cm span^59^.

### Bone mineral density

The bone mineral density (BMD) of all the collected tibiae was analyzed using quantitative computed tomography (QCT) at the college of Veterinary Medicine, Konkuk University (Korea, Seoul). Three positions of each tibia including neck (section of the mastoid arthrodesis), 1/3 of the proximal portion, and 2/3 of the distal portion were scanned using a CT scanner (LightSpeed Plus, GE Healthcare, Amersham, UK). The scanning conditions were as follows: 120 kV and 200 mA, slice thickness 1.25 mm, slice interval 1.25 mm, pitch 1.5:1, rotation time 0.6 s, and scanning speed 7.5 mm/rotation. The scanned images were archived in DICOM (Digital Imaging and COmmunications in Medicine) format and were evaluated by using a 3D slicer software (Version 4.6.2 r25516, National Alliance for Medical Image Computing). The Hounsfield unit (HU) values of each standard point of the three positions in a tibia were taken and the trend equation was obtained using a single-layer computerized photograph through QCT calibration phantom (QRM-BDC/3, QRM GmbH, Moehrendorf, Germany). The HU values obtained from the QCT scans were used to calculate BMD (mg/cm^3^).

### Statistical analysis

Data were analyzed in a completely randomized design with 5 treatments using the PROC GLM procedures of SAS 9.4 (SAS Institute, Cary, NC, USA). The replicate (9 hens each) was the experimental unit for analysis of performance data. The egg quality traits were analyzed statistically at each week by considering the number of eggs as the experimental unit. The data were pooled for all 7-week periods and the results are presented in the manuscript. For blood parameters and bone quality measurements, the individual bird served as the experimental unit. Significant differences among the treatments were determined using Duncan’s multiple range test at *P* < 0.05. Effects of the ES particle size (LS, ESF, and ESC) were further determined by the “contrast” option of the GLM procedure. Data are presented as the least squares means and standard error of the means (SEM).

## Supplementary Information

Supplementary Tables.

## Data Availability

The data analyzed during the current study are available from the corresponding author on reasonable request.
