# Supplementary material for: Superiority of coarse eggshell as a calcium source over limestone, cockle shell, oyster shell, and fine eggshell in old laying hens
Source: Sci Rep. 2021 Jun 24;11:13225. doi: 10.1038/s41598-021-92589-y (PMC8225885; doi:10.1038/s41598-021-92589-y)
Supplement: Supplementary file 1 — Supplementary Tables. [file 41598_2021_92589_MOESM1_ESM.docx]

**Supplementary data**

**Superiority of coarse eggshell as a calcium source over limestone, cockle shell, oyster shell, and fine eggshell in old laying hens**

**Woo-Do Lee^*, †^, Damini Kothari^*, †^, Kai-Min Niu^*, ‡^, Jeong-Min Lim^*^, Da-Hye Park^*^, Jaeeun Ko^§^, Kidong Eom^§^, and Soo-Ki Kim^*, 1^**

**^*^***Department of Animal Science and Technology, Konkuk University, 120 Neungdong-ro, Gwangjin-gu, Seoul 05029, Republic of Korea*

**^‡^** *Institute of Biological Resource, Jiangxi Academy of Sciences, Nanchang 330029, China*

*^§^ Department of Veterinary Radiology and Diagnostic Imaging, College of Veterinary Medicine, Konkuk University, 120 Neungdong-ro, Gwangjin-gu, Seoul 05029, Republic of Korea*

^†^The authors contributed equally to this study.

**^1^Corresponding Author**:

**Soo-Ki Kim**

Department of Animal Science and Technology

Konkuk University, Seoul 05029, Republic of Korea

Tel: +82-2-450-3728; Fax: +82-2-458-3728

E-mail: [sookikim@konkuk.ac.kr](mailto:sookikim@konkuk.ac.kr)

**Table S1.** Egg quality traits in laying hens fed with different dietary Ca from 73 to 80 wk of age.

| **Items** | **Treatments^1)^** | | | | | **SEM^2)^** | ***P*-value** | |
| --- | --- | --- | --- | --- | --- | --- | --- | --- |
|  | **LS** | **CS** | **OS** | **ESF** | **ESC** |  | **Source** | **ES size** |
| Egg weight, g |  |  |  |  |  |  |  |  |
| 1 wk | 64.91 | 62.99 | 64.68 | 65.19 | 64.38 | 0.388 | 0.421 | 0.508 |
| 2 wk | 64.33^b^ | 61.85^c^ | 64.82^ab^ | 64.94^ab^ | 67.08^a^ | 0.404 | 0.002 | 0.085 |
| 3 wk | 65.07 | 63.19 | 64.64 | 65.29 | 65.14 | 0.363 | 0.349 | 0.899 |
| 4 wk | 65.78 | 63.01 | 65.36 | 65.24 | 66.75 | 0.402 | 0.051 | 0.229 |
| 5 wk | 65.28^abc^ | 63.58^c^ | 64.68^bc^ | 66.36^ab^ | 67.53^a^ | 0.397 | 0.018 | 0.342 |
| 6 wk | 66.47^a^ | 63.22^b^ | 65.50^ab^ | 65.51^ab^ | 67.13^a^ | 0.406 | 0.030 | 0.202 |
| 7 wk | 65.96 | 64.20 | 64.85 | 65.49 | 66.10 | 0.359 | 0.419 | 0.587 |
|  |  |  |  |  |  |  |  |  |
| Haugh units |  |  |  |  |  |  |  |  |
| 1 wk | 85.96 | 85.83 | 85.38 | 85.59 | 87.15 | 0.589 | 0.897 | 0.407 |
| 2 wk | 86.66 | 84.65 | 87.61 | 84.59 | 86.05 | 0.467 | 0.182 | 0.323 |
| 3 wk | 86.23^b^ | 84.38^b^ | 89.10^a^ | 86.16^b^ | 87.28^ab^ | 0.441 | 0.014 | 0.413 |
| 4 wk | 84.42 | 85.20 | 86.47 | 85.90 | 87.35 | 0.409 | 0.191 | 0.262 |
| 5 wk | 85.62^a^ | 81.38^b^ | 84.36^a^ | 85.64^a^ | 86.23^a^ | 0.429 | 0.002 | 0.647 |
| 6 wk | 83.40^b^ | 82.57^b^ | 86.76^a^ | 84.51^ab^ | 84.67^ab^ | 0.361 | 0.004 | 0.889 |
| 7 wk | 82.05 | 81.45 | 83.43 | 81.81 | 83.00 | 0.399 | 0.481 | 0.345 |
|  |  |  |  |  |  |  |  |  |
| Albumen height, mm |  |  |  |  |  |  |  |  |
| 1 wk | 7.70 | 7.51 | 7.67 | 7.89 | 7.84 | 0.090 | 0.687 | 0.862 |
| 2 wk | 7.64 | 7.21 | 7.93 | 7.50 | 7.61 | 0.079 | 0.633 | 0.641 |
| 3 wk | 7.60^ab^ | 7.27^b^ | 8.10^a^ | 7.70^ab^ | 7.86^a^ | 0.076 | 0.009 | 0.504 |
| 4 wk | 7.34^b^ | 7.35^b^ | 7.77^ab^ | 7.57^ab^ | 7.89^a^ | 0.071 | 0.046 | 0.151 |
| 5 wk | 7.64^a^ | 6.87^b^ | 7.26^ab^ | 7.53^a^ | 7.69^a^ | 0.071 | < 0.001 | 0.460 |
| 6 wk | 6.88^d^ | 7.03^cd^ | 7.77^a^ | 7.43^ab^ | 7.35^bc^ | 0.062 | < 0.001 | 0.664 |
| 7 wk | 6.99 | 6.73 | 7.08 | 6.85 | 7.13 | 0.061 | 0.211 | 0.155 |
|  |  |  |  |  |  |  |  |  |
| Egg shell color |  |  |  |  |  |  |  |  |
| 1 wk | 39.78 | 38.28 | 39.08 | 38.95 | 39.73 | 0.309 | 0.527 | 0.425 |
| 2 wk | 35.00^a^ | 33.93^a^ | 33.50^ab^ | 34.00^a^ | 32.38^b^ | 0.238 | 0.011 | 0.028 |
| 3 wk | 34.43 | 33.30 | 33.60 | 33.38 | 34.45 | 0.197 | 0.160 | 0.085 |
| 4 wk | 33.40 | 33.70 | 33.48 | 33.35 | 33.60 | 0.187 | 0.414 | 0.206 |
| 5 wk | 34.10 | 34.00 | 34.70 | 34.28 | 33.98 | 0.232 | 0.860 | 0.685 |
| 6 wk | 33.30^a^ | 33.63^a^ | 33.95^a^ | 31.78^b^ | 33.18^a^ | 0.176 | 0.001 | 0.010 |
| 7 wk | 34.83^ab^ | 33.93^b^ | 35.50^a^ | 33.85^b^ | 33.75^b^ | 0.183 | 0.007 | 0.860 |
|  |  |  |  |  |  |  |  |  |
| Egg yolk color |  |  |  |  |  |  |  |  |
| 1 wk | 5.98 | 5.82 | 5.83 | 5.94 | 5.79 | 0.053 | 0.742 | 0.376 |
| 2 wk | 5.76 | 5.72 | 5.78 | 5.96 | 6.01 | 0.040 | 0.076 | 0.650 |
| 3 wk | 6.12^a^ | 5.81^b^ | 5.79^b^ | 6.04^ab^ | 5.96^ab^ | 0.041 | 0.043 | 0.521 |
| 4 wk | 5.65^bc^ | 5.55^c^ | 5.63^bc^ | 5.98^a^ | 5.85^ab^ | 0.036 | < 0.001 | 0.217 |
| 5 wk | 5.64^c^ | 5.66^c^ | 5.76^bc^ | 6.10^a^ | 5.89^b^ | 0.033 | < 0.001 | 0.037 |
| 6 wk | 5.66^b^ | 5.72^b^ | 5.91^a^ | 5.95^a^ | 5.97^a^ | 0.029 | < 0.001 | 0.801 |
| 7 wk | 5.66 | 5.70 | 5.55 | 5.81 | 5.71 | 0.028 | 0.075 | 0.280 |
|  |  |  |  |  |  |  |  |  |
| Egg shell breaking strength, kgf |  |  |  |  |  |  |  |  |
| 1 wk | 3.49 | 3.82 | 3.63 | 3.41 | 3.65 | 0.070 | 0.380 | 0.283 |
| 2 wk | 3.69 | 3.75 | 3.80 | 3.96 | 3.79 | 0.058 | 0.673 | 0.356 |
| 3 wk | 3.60 | 3.80 | 3.74 | 3.88 | 3.83 | 0.058 | 0.614 | 0.786 |
| 4 wk | 3.52 | 3.95 | 3.86 | 3.92 | 3.62 | 0.058 | 0.065 | 0.107 |
| 5 wk | 3.46^b^ | 3.97^a^ | 3.58^b^ | 3.62^b^ | 3.76^ab^ | 0.055 | 0.036 | 0.395 |
| 6 wk | 3.81 | 3.65 | 3.84 | 3.78 | 3.94 | 0.050 | 0.506 | 0.312 |
| 7 wk | 3.78 | 3.61 | 3.63 | 3.67 | 3.75 | 0.049 | 0.764 | 0.594 |
|  |  |  |  |  |  |  |  |  |
| Egg shell thickness, mm |  |  |  |  |  |  |  |  |
| 1 wk | 0.394 | 0.401 | 0.410 | 0.399 | 0.399 | 0.002 | 0.376 | 0.975 |
| 2 wk | 0.387 | 0.381 | 0.397 | 0.368 | 0.391 | 0.003 | 0.059 | 0.030 |
| 3 wk | 0.372 | 0.368 | 0.372 | 0.363 | 0.380 | 0.003 | 0.294 | 0.034 |
| 4 wk | 0.404^a^ | 0.414^a^ | 0.407^a^ | 0.380^b^ | 0.382^b^ | 0.002 | < 0.001 | 0.807 |
| 5 wk | 0.405 | 0.413 | 0.4106 | 0.405 | 0.404 | 0.002 | 0.532 | 0.845 |
| 6 wk | 0.387 | 0.387 | 0.377 | 0.387 | 0.384 | 0.002 | 0.467 | 0.728 |
| 7 wk | 0.394 | 0.389 | 0.384 | 0.383 | 0.381 | 0.002 | 0.281 | 0.771 |

^1)^LS: Limestone (<2 and 2-4 mm mixed in 3:7); CS: Cockle shell (1-4 mm); OS: Oyster shell (3-16 mm); ESF: Eggshell fine particles (<1 mm); ESC: Eggshell coarse particles (3-5 mm).

^2)^SEM: Standard error of means (n=40).

ES: Eggshell

^a-d^Means with the different superscript in the same row differ significantly (*P* < 0.05).

**Table S2.** Blood biochemical constituents of laying hens at 80 wk of age according to the treatments.

| **Items** | **Treatments^1)^** | | | | | **SEM^2)^** | ***P*-value** | |
| --- | --- | --- | --- | --- | --- | --- | --- | --- |
|  | **LS** | **CS** | **OS** | **ESF** | **ESC** |  | **Source** | **ES size** |
| **3 pm** |  |  |  |  |  |  |  |  |
| AST, U/L | 159.8 | 164.0 | 162.8 | 186.8 | 211.7 | 6.57 | 0.083 | 0.218 |
| ALT, U/L | 8.50 | 3.50 | 5.00 | 5.25 | 7.25 | 1.05 | 0.631 | 0.570 |
| LDH, mg/dl | 5455.0 | 6718.0 | 7618.0 | 7183.0 | 7483.0 | 381.4 | 0.429 | 0.820 |
| TG, mg/dl | 1740.0 | 1157.5 | 2425.0 | 2455.0 | 2625.0 | 254.1 | 0.337 | 0.831 |
| TC, mg/dl | 105.3 | 80.0 | 135.0 | 125.3 | 124.3 | 7.78 | 0.171 | 0.965 |
| HDL, mg/dl | 41.50 | 31.25 | 32.50 | 39.00 | 33.00 | 1.68 | 0.219 | 0.249 |
| HDL, % | 40.74^a^ | 39.84^a^ | 23.68^b^ | 32.13^ab^ | 27.89^b^ | 2.09 | 0.017 | 0.420 |
| LDL+VLDL, mg/dl | 63.75 | 48.75 | 102.50 | 86.25 | 91.25 | 7.17 | 0.097 | 0.806 |
| Glucose, mg/dl | 253.3 | 292.5 | 254.8 | 265.8 | 260.0 | 9.32 | 0.740 | 0.868 |
| TP, g/dl | 5.83^bc^ | 5.00^c^ | 6.85^ab^ | 6.35^ab^ | 7.00^a^ | 0.21 | 0.004 | 0.179 |
| Albumin, g/dl | 1.85^b^ | 1.75^b^ | 1.98^b^ | 1.83^b^ | 2.48^a^ | 0.08 | 0.018 | 0.006 |
| Creatinine, mg/dl | 0.200^b^ | 0.275^ab^ | 0.275^ab^ | 0.250^b^ | 0.375^a^ | 0.019 | 0.037 | 0.023 |
| BUN, mg/dl | 2.33^b^ | 2.43^b^ | 2.53^b^ | 2.48^b^ | 2.95^a^ | 0.06 | 0.005 | 0.004 |
| **9 pm** |  |  |  |  |  |  |  |  |
| AST, U/L | 197.8 | 200.3 | 181.0 | 187.0 | 226.8 | 9.01 | 0.592 | 0.196 |
| ALT, U/L | 4.75 | 5.25 | 5.50 | 5.00 | 5.75 | 0.32 | 0.895 | 0.500 |
| LDH, mg/dl | 7210.0 | 9420.0 | 6735.0 | 4600.0 | 5370.0 | 599.7 | 0.082 | 0.647 |
| TG, mg/dl | 2832.5 | 2342.5 | 2467.5 | 3142.5 | 3775.0 | 275.4 | 0.518 | 0.486 |
| TC, mg/dl | 141.0 | 117.5 | 135.0 | 155.8 | 187.5 | 10.04 | 0.246 | 0.309 |
| HDL, mg/dl | 39.00 | 34.75 | 34.50 | 39.75 | 43.25 | 1.31 | 0.166 | 0.371 |
| HDL, % | 29.03 | 30.13 | 25.50 | 25.86 | 25.54 | 1.05 | 0.402 | 0.695 |
| LDL+VLDL, mg/dl | 102.0 | 82.8 | 100.5 | 116.0 | 144.3 | 8.94 | 0.271 | 0.313 |
| Glucose, mg/dl | 283.8 | 272.5 | 280.8 | 226.3 | 290.3 | 15.76 | 0.756 | 0.245 |
| TP, g/dl | 6.93 | 7.33 | 7.18 | 7.08 | 7.43 | 0.11 | 0.722 | 0.381 |
| Albumin, g/dl | 2.70 | 2.45 | 2.25 | 2.25 | 2.45 | 0.12 | 0.731 | 0.596 |
| Creatinine, mg/dl | 0.275 | 0.300 | 0.300 | 0.275 | 0.175 | 0.027 | 0.626 | 0.284 |
| BUN, mg/dl | 2.35 | 2.63 | 2.45 | 2.70 | 2.35 | 0.07 | 0.472 | 0.157 |
| **3 am** |  |  |  |  |  |  |  |  |
| AST, U/L | 196.5 | 185.0 | 170.5 | 161.0 | 148.5 | 10.31 | 0.651 | 0.718 |
| ALT, U/L | 6.50 | 4.00 | 6.50 | 5.25 | 13.25 | 1.92 | 0.639 | 0.226 |
| LDH, mg/dl | 7583.0 | 6398.0 | 4890.0 | 5000.0 | 6243.0 | 601.0 | 0.647 | 0.540 |
| TG, mg/dl | 2820.0 | 2200.0 | 3040.0 | 3655.0 | 2998.0 | 330.3 | 0.778 | 0.563 |
| TC, mg/dl | 155.8 | 114.0 | 144.3 | 169.8 | 161.5 | 13.95 | 0.789 | 0.863 |
| HDL, mg/dl | 39.50 | 31.25 | 34.75 | 38.25 | 33.50 | 2.49 | 0.860 | 0.584 |
| HDL, % | 26.49 | 28.12 | 24.42 | 24.16 | 22.18 | 2.49 | 0.583 | 0.606 |
| LDL+VLDL, mg/dl | 116.3 | 82.75 | 109.5 | 131.5 | 128.0 | 1.16 | 0.756 | 0.931 |
| Glucose, mg/dl | 261.3 | 276.8 | 313.3 | 317.0 | 262.8 | 13.15 | 0.533 | 0.220 |
| TP, g/dl | 7.68 | 5.78 | 6.30 | 6.88 | 6.90 | 0.23 | 0.095 | 0.970 |
| Albumin, g/dl | 2.75 | 1.73 | 1.85 | 2.30 | 3.03 | 0.23 | 0.353 | 0.333 |
| Creatinine, mg/dl | 0.275 | 0.175 | 0.225 | 0.200 | 0.275 | 0.025 | 0.688 | 0.384 |
| BUN, mg/dl | 2.65 | 2.15 | 2.25 | 2.75 | 2.30 | 0.10 | 0.282 | 0.174 |

^1)^LS: Limestone (< 2 and 2-4 mm mixed in 3:7); CS: Cockle shell (1-4 mm); OS: Oyster shell (3-16 mm); ESF: Eggshell fine particles (<1 mm); ESC: Eggshell coarse particles (3-5 mm).

^2)^SEM: Standard error of means (n=4).

ES: Eggshell

^a-d^Means with the different superscript in the same row differ significantly (*P* < 0.05).

**Table S3.** Changes in the serum Ca and P levels during light and dark periods (18 h) in laying hens fed with different dietary Ca at 80 wk of age.

| **Items** | **Treatments^1)^** | | | | | **SEM^2)^** | ***P*-value** | |
| --- | --- | --- | --- | --- | --- | --- | --- | --- |
|  | **LS** | **CS** | **OS** | **ESF** | **ESC** |  | **Source** | **ES size** |
| 0 h (3 pm) |  |  |  |  |  |  |  |  |
| Ca, mg/dl | 30.40 | 26.43 | 33.80 | 32.25 | 30.10 | 1.09 | 0.278 | 0.524 |
| P, mg/dl | 7.4 | 6.7 | 7.8 | 7.8 | 7.9 | 0.305 | 0.765 | 0.924 |
|  |  |  |  |  |  |  |  |  |
| 6 h (9 pm) |  |  |  |  |  |  |  |  |
| Ca, mg/dl | 30.10 | 30.55 | 36.90 | 32.73 | 34.70 | 1.17 | 0.335 | 0.594 |
| P, mg/dl | 7.40 | 7.30 | 8.08 | 8.05 | 7.98 | 0.246 | 0.795 | 0.929 |
|  |  |  |  |  |  |  |  |  |
| 12 h (3 am) |  |  |  |  |  |  |  |  |
| Ca, mg/dl | 34.38 | 28.25 | 30.73 | 32.73 | 30.58 | 1.48 | 0.783 | 0.673 |
| P, mg/dl | 9.58 | 7.50 | 8.10 | 9.08 | 7.48 | 0.429 | 0.625 | 0.649 |
|  |  |  |  |  |  |  |  |  |
| 18 h (9 am) |  |  |  |  |  |  |  |  |
| Ca, mg/dl | 31.63 | 27.78 | 28.70 | 29.73 | 30.55 | 1.14 | 0.875 | 0.836 |
| P, mg/dl | 7.83 | 6.85 | 5.43 | 6.05 | 6.18 | 0.433 | 0.508 | 0.930 |

^1)^LS: Limestone (< 2 and 2-4 mm in 3:7); CS: Cockle shell (1-4 mm); OS: Oyster shell (3-6 mm); ESF: Eggshell fine particles (< 1 mm); ESC: Eggshell coarse particles (3-5 mm).

^2)^SEM: Standard error of means (n=4)

^a-d^Means with the different superscript in the same row differ significantly (*P* < 0.05).

**Table S4.** Ingredients and nutrient composition of the commercial diet.

| **Items** | **Amount** |
| --- | --- |
| Ingredients, % |  |
| Corn | 53.8 |
| Soybean meal | 22.9 |
| Rapeseed meal | 2.50 |
| Distillers dried grains with solubles | 8.00 |
| Tallow | 0.800 |
| Methionine (98%) | 0.125 |
| Dicalcium phosphate | 0.500 |
| Choline-Cl (liquid) 50% | 10.7 |
| Limestone, 37.2% Ca^1)^ | 0.250 |
| Salt | 0.125 |
| Sodium bicarbonate | 0.110 |
| Vitamin premix^2)^ | 0.100 |
| Mineral premix^3)^ | 0.050 |
| Phytase^4)^ | 0.085 |
|  | 100 |
| Nutrient composition, % | |
| Crude protein | 17.0 |
| Crude fat | 3.80 |
| Crude fiber | 2.80 |
| Ash | 14.2 |
| Ca | 4.10 |
| Total P | 0.440 |
| Met+Cys^5)^ | 0.700 |
| Metabolizable energy (MJ/kg) | 11.4 |

^1)^Particle size: < 2 and 2-4 mm mixed in 3:7.

^2)^Vitamin premix supplied the following per kg of diet: vitamin A, 8,000 IU; vitamin D3, 3,300 IU; vitamin E, 20 g; vitamin K, 2.5 g; vitamin B1, 2.5 g; vitamin B2, 5.5 g; vitamin B3, 30 g; vitamin B5, 8 g; vitamin B6, 4 g; vitamin B7, 75 mg, vitamin B9, 0.9 g; vitamin B12, 23 mg.

^3)^Mineral premix supplied the following per kg of diet: Choline, 110 g; Manganese, 90 g; Zinc, 80 g; Iron, 40 g; Copper, 8 g; Iodine, 1.2 g; Selenium, 0.22 g.

^4)^1,000,000 phytase units (FTU).

^5)^Met: Methionine; Cys: Cysteine.
